# 2265 Effect of dietary approaches to stop hypertension (DASH) diet on hemodynamic markers in advanced heart failure patients

**DOI:** 10.1017/cts.2018.166

**Published:** 2018-11-21

**Authors:** Elisabeth L. P. Sattler, Sandra B. Dunbar, Arshed A. Quyyumi, Jonathan R. Murrow, Richard D. Lewis, Henry N. Young, Whitni McConnell

**Affiliations:** Emory University

## Abstract

OBJECTIVES/SPECIFIC AIMS: The central aim of the study is to examine the effect of a Dietary Approaches to Stop Hypertension (DASH) diet on hemodynamic, cardiometabolic, and inflammatory markers in advanced heart failure patients with implanted hemodynamic monitoring devices. METHODS/STUDY POPULATION: This pilot study will employ a clinical feeding trial using a 1-group pre-post test design with an anticipated sample size of n=36 (n=20 plus 44% expected attrition). Heart failure patients 18+ years of age with English language literacy, classified as NYHA functional stage III, regardless of ventricular ejection fraction, who have undergone CardioMEMS™ hemodynamic monitoring device (St. Jude Medical, Atlanta, GA, USA) implantation and have received optimized heart failure therapy for 3+ months will be recruited at Piedmont Athens Regional Hospital in Athens, GA. The study is divided in (a) a calibration (self-selected diet) and (b) a DASH feeding intervention phase (each 21 days in length). The DASH meals will strictly follow meal planning guidelines published by the National Heart, Lung, and Blood Institute of the National Institutes of Health, and be prepared under the supervision of a registered dietitian at the University Health Center in Athens, GA. The DASH diet is a heart-healthy eating pattern that is focused on adequate consumption of fruits, vegetables, whole grains, low-fat dairy, fish, poultry, beans, nuts, and vegetables oils while emphasizing limited intake of foods containing saturated fat, such as fatty red meats, full-fat dairy products, and tropical oils, such as coconut, palm kernel, and palm oils, as well as sugar-sweetened beverages and sweets. Participants will visit the University of Georgia Clinical and Translational Research Unit on 3 occasions at baseline, upon completion of the calibration phase, and following completion of the intervention phase for repeated collection of anthropometric (height, weight, waist and hip circumference, percent body fatness), cardiometabolic (blood pressure, blood glucose, HbA1C, lipid panel, basic metabolic panel, BNP, NT-proBNP, troponin 1, MR-proADM, sST2), functional status (6-min walk test), inflammatory (IL-1a, IL-1b, IL-6, TNF-a), and self-reported measures (demographic and economic characteristics, health, chronic diseases, perceived stress, heart failure-related quality of life, social support, sleep quality, food insecurity, tobacco smoking status, healthcare utilization, medication adherence). Hemodynamic marker (pulmonary artery pressure, heart rate) and pharmacotherapy information (medication count, type, strength, and dosing) will be obtained from through retrospective assessment of EHR data. Descriptive statistics [percentage, mean (SD), median (IQR), mode, range] will be used to describe sample characteristics at each of the study visits, as well as characteristics of participants’ self-selected diets during the calibration phase. To measure changes in hemodynamic, cardiometabolic, and inflammatory markers pre-post DASH diet intervention, we will use paired Student *t*-tests (normal distribution) or Wilcoxon rank-sum tests (non-normal distribution), as appropriate. Data collection will be carried out between February and November 2018. RESULTS/ANTICIPATED RESULTS: The study builds upon previous studies showing improvement of ventricular function, arterial stiffness, oxidative stress, and blood pressure after short-term consumption of a sodium-restricted DASH diet in heart failure patients with preserved ejection fraction, and will provide new information on the cumulative effect of short-term adherence with a DASH diet on indicators of heart failure complications, including hemodynamic, cardiometabolic, and inflammatory markers. In addition, it will give better insight on heart failure patients’ habitual dietary intake in the context of other sociodemographic, economic, health, and social factors. DISCUSSION/SIGNIFICANCE OF IMPACT: Findings from the proposed study will provide key knowledge of dietary influences on ventricular function in order to define evidence-based diet therapy needed for the early prevention of HF complications in advanced heart failure patients.

